# The status and use of prosthetic devices by persons with lower limb amputation in Rwanda

**DOI:** 10.4102/ajod.v11i0.1081

**Published:** 2022-12-09

**Authors:** Robert Ngarambe, Jean Baptiste Sagahutu, Assuman Nuhu, David K. Tumusiime

**Affiliations:** 1 Department of Physiotherapy, Faculty of Health Sciences, University of Rwanda, Kigali, Rwanda; 2 Department of Rehabilitation, The Regional Centre of Excellence in Biomedical Engineering and eHealth, University of Rwanda, Kigali, Rwanda

**Keywords:** admitted, experiences, family members, relative, state patient, qualitative

## Abstract

**Background:**

Amputation is one of the leading causes of disabilities because of reduced mobility. Without assistive devices specifically prostheses, the quality of life of persons with lower limb amputation (PLLA) further deteriorates. Therefore, prostheses are fundamental to improving their quality of life.

**Objectives:**

This study aimed to establish the number of PLLA with or without prosthesis and to determine their socio-economic profile in Rwanda.

**Method:**

A descriptive, cross-sectional study was conducted in all sectors of Rwanda. As a result of coronavirus disease 2019 movement restrictions, data collection was carried out through telephone calls with participants to complete the questionnaires. Descriptive, inferential statistics and chi-square test were performed to analyse data using Statistical Package for Social Science (SPSS) 21.0.

**Results:**

Of the 3026 participants identified countrywide, 68.8% were males and 60.3% of them did not have any prosthesis (*p* = 0.003). The majority (62.4%) of those who had prosthetic devices needed repair of their prostheses while 14.8% of participants reported that their prosthetic devices were completely broken and/or damaged (*p* = 0.604). Among the participants, 63.7% had no source of income and 66.7% had dependents (*p ≤* 0.001).

**Conclusion:**

The majority of the PLLA in Rwanda did not have prosthetic devices and even those with prostheses did not fully function and thus required repair. Therefore, it adversely affects their livelihood.

**Contribution:**

The government should collaborate with stakeholders working with persons with disabilities and implement mechanisms and/or strategies to make prosthetic devices accessible and affordable.

## Introduction

Loss of a body limb leads to reduced functioning that restricts the individual’s participation in the community (Van Twillert et al. 2014). Amputation of a part or whole limb causes permanent disability leading to changes in functioning in life (Knežević et al. 2015). It is estimated, globally, that approximately 73.5% of limb amputations are lower limbs. The major causes of amputation are traumatic injuries and vascular disorders (Asano et al. 2008). In high-income countries, vascular disorders are the main cause of amputations, while in low-income countries, traumatic injuries are the major cause of amputation (Sinha, Van Den Heuvel & Arokiasamy 2011). Diabetes and vascular disorders are increasingly becoming a health concern in low-income countries, hence leading to amputation (Agu & Ojiaku 2016; Ahmad et al. 2019; Sangam et al. 2015).

In Rwanda, the number of persons with physical disabilities, including those with lower limb amputation (LLA) is estimated at about 5% of the general population, of which 1.6% had LLA according to the national census of 2012 (National Institute of Statistics of Rwanda 2014). According to the disability categorisation process of 2016 findings, approximately 88% of persons with lower limb amputation (PLLA) needed prostheses (Kidd & Kabare 2019). However, the provision of prostheses to PLLA seems to be costly because of the high cost of production and procurement of raw materials, as well as additional costs of transport to rehabilitation centres (Matter & Eide 2018; Rhoda & Eide 2009).

Lower limb amputees without prostheses as mobility assistive devices have increasingly had an impact on their lives, such as the decline of physical functioning and quality of life at the individual, family and society levels (Ng et al. 2020). Without lower limb prostheses, PLLA are often excluded and locked into persistent poverty and isolation (Anderson, Kaiser Gladwin & Mayo 2016). However, if PLLA are given prosthetic devices and rehabilitated back to full functional capacity to carry out daily activities and participate actively and productively in community life (Smith et al. 2018a), this may reduce dependence on both community and their families, hence improving quality of life. The aim of this study was to establish the number of PLLA with or without mobility assistive devices or prosthesis and to determine their socio-economic profile in Rwanda.

## Research methods and design

A cross-sectional and descriptive study design was conducted in Rwanda. The accumulative census on PLLA was carried out in the 416 sectors across the country. Participants were contacted through the local authorities at the cell level, to request them to participate in the survey. Regarding the inclusion criterion for participating in this survey any person with LLA with or without a prosthesis at any age was eligible.

Prior to data collection, research assistants were trained by the researcher on the data collection instrument, the aim of the study, the data collection procedure, as well as ethical considerations. Data from the participants were collected by research assistants through telephone interviews to complete the questionnaire. The questionnaire was an adapted section of the Trinity Amputation and Prosthetic Experiences Scale (TAPES-R), and another section from the World Health Organization Disability Assessment Schedule (WHODAS-02-Demographic and background information) used in a similar setting in Rwanda (Scorza et al. 2013). Both are standardised instruments and their validity and reliability were tested. World Health Organization Disability Assessment Schedule 2.0: Cronbach’s α coefficient was 0.96 and Pearson’s correlation coefficient was 0.98 (Üstün et al. 2010). Trinity Amputation and Prosthetic Experiences Scale-R: Test–retest reliability ranged from 0.66 to 0.87 (Gallagher & MacLachlan 2000). These questionnaires were adapted, in this study, to suit the Rwandan context. The questionnaires were translated from English to Kinyarwanda by two language experts and back to English by two other language experts to address the cultural and linguistic equivalence. Then, the questionnaire was sent to experts in the field of rehabilitation for their opinion on the quality of translation, clarity and suitability for the Rwandan participants.

The questionnaire was composed of 18 items divided into three sections (demographic, amputation profile and socio-economic sections). The National Council of Persons with Disabilities, in Rwanda, has a formal structure from the national to the community level. Therefore, the researcher contacted the in charge of persons with disabilities at the district level, who then consulted with the coordinator of persons with disabilities in the community to get cell phone numbers for all PLLA or their caregivers. After getting the contact numbers, the participants were first approached on their cell phones by research assistants who explained to them in detail the purpose of the study and requested their voluntary participation. Then, data were collected through phone calls from all those who verbally consented to complete the questionnaire. Data were analysed using the Statistical Package for Social Science (SPSS) version 21.0. Descriptive statistics were performed to summarise the demographic data. Chi-square tests were performed to determine the association between amputation profiles and other variables such as demographic data, the status of a prosthesis and socio-economic sections (source of income, social-economic category and dependents). The level of significance was set at *p* < 0.05.

### Ethical considerations

The study was approved for ethical clearance by the Institution Review Board (IRB) of the University of Rwanda, College of Medicine and Health Sciences; N°369/CMHSIRB/2020.

## Results

### Socio-demographic characteristics of participants

Of the 3362 persons with LLA, 3026 persons participated in this study, which was equivalent to 1.6% among persons with disabilities in Rwanda (National Institute of Statistics of Rwanda 2014). The participants’ age ranged from 3 to 101 years, with a mean age of 49.1 years (standard deviation [SD] = 18.5). Among the participants, males accounted for 68.8% (*n* = 2081) and 31.2% (*n* = 945) females (*p* = 0.003). Among the participants, 39.7% (*n* = 1202) had prosthetic devices and 60.3% (*n* = 1824) did not have prosthetic devices. As highlighted in Table 1, a statistically significant association was found between gender and possession of prosthetic devices among persons with LLA (*p* = 0.003).

**TABLE 1:** Socio-demographic characteristics of participants.

Demographics	Possession of prosthesis				Total		*p*
	No		Yes				
	*n*	%	*n*	%	*N*	%	
**Gender**	-	-	-	-	-	-	0.003
Male	1217	40.2	864	28.6	2081	68.8	-
Female	607	20.1	338	11.2	945	31.2	-
**Level of education**	-	-	-	-	-	-	< 0.001
No education level	606	20.0	164	5.4	770	25.4	-
Primary level	884	29.2	589	19.5	1473	48.7	-
Secondary level	270	8.9	329	10.9	599	19.8	-
Vocational school	47	1.6	60	2.0	107	3.5	-
University	13	0.4	60	2.0	73	2.4	-
N.A.	4	0.1	0	0.0	4	0.1	-
**Marital status**	-	-	-	-	-	-	< 0.001
Single	300	9.9	271	9.0	571	18.9	-
Married	928	30.7	675	22.3	1603	53.0	-
Separated	41	1.4	26	0.9	67	2.2	-
Divorced	73	2.4	30	1.0	103	3.4	-
Widowed	212	7.0	83	2.7	295	9.7	-
Cohabiting	185	6.1	82	2.7	267	8.8	-
N.A.	85	2.8	35	1.2	120	4.0	-
**Residence**	-	-	-	-	-	-	< 0.001
Urban	286	9.5	273	9.0	559	18.5	-
Rural	1538	50.8	929	30.7	2467	81.5	-
**Social class Ubudehe**	-	-	-	-	-	-	< 0.001
Category 1	1261	41.7	607	20.1	1868	61.7	-
Category 2	516	17.1	473	15.6	989	32.7	-
Category 3	47	1.6	122	4.0	169	5.6	-

**Total**	**1824**	**60.3**	**1202**	**39.7**	**3026**	**100.0**	-

N.A., not applicable.

Regarding participants’ level of education, those with primary level were 48.7%; (*n* = 1473), of which 19.5% (*n* = 589) reported that they had prosthetic devices. The education level with the least participants was university level, 2.4% (*n* = 73) from which 2%; (*n* = 60) had prosthetic devices (*p ≤* 0.001). There was a statistically significant association between the level of education and possession of prosthetic devices (*p ≤* 0.001). Findings on participants’ marital status revealed that 53% (*n* = 1603) were married of which 22.3% (*n* = 675) had prosthetic devices and the divorced were 3.4% (*n* = 103) of which 1% had prosthetic devices. There was a statistically significant association between marital status and possession of prosthetic devices (*p ≤* 0.001). The majority (81.5%; *n* = 2467) of PLLA lived in a rural area, of which 30.7% (*n* = 929) had prosthetic devices. Participants in urban areas 9.0% (*n* = 273) had prosthetic devices. There was a statistically significant association between possession of a prosthetic device and area of residence (*p ≤* 0.001). Among the participants, 61.7% (*n* = 1868) were in Category 1 of the social-economic status, of which 20.1% (*n* = 607) had prosthetic devices, 5.6% (*n* = 169) of the participants were in Category 3 of which 4.0% (*n* = 122) had prosthetic devices. A statistically significant association was found between the socio-economic status (Ubudehe categories) and possession of prosthetic devices (*p ≤* 0.001) (Table 1).

The Ubudehe category is a Rwanda government classification method of the population according to socio-economic status. The categories are divided into four parts: Category 1 is for the very poor, Category 2 is for the relatively poor, Category 3 is for the relatively wealthy and Category 4 is for the wealthy population.

### Socio-economic characteristics of participants with or without prosthetic devices

Furthermore, 46.4% (*n* = 1405) of participants were unemployed, of whom 14.7% (*n* = 445) had prosthetic devices and 4.8% (*n* = 145) of the participants had paid work, of whom 3.3% (*n* = 99) had prosthetic devices (Table 2). A statistically significant association was found between employment and possession of prosthetic devices (*p ≤* 0.001). The study shows that 47.1% (*n* = 1426) of the participants had below the knee amputation; among these participants 22% (*n* = 667) had prosthetic devices while above the knee amputations were 45.7% (*n* = 1383) of whom 16.6% (*n* = 503) had prosthetic devices. There is a statistically significant association between the level of amputation and possession of prosthetic devices. Among participants with LLA, 62.9% (*n* = 1903) had dependents and among them, 26.9% (*n* = 814) had prosthetic devices. A total of 67.2% (*n* = 2032) of the participants did not have financial assistance; however, 29% (*n* = 879) of them had prosthetic devices. Of the 32.8% (*n* = 994) with financial assistance, only 10.7% had prosthetic devices. A statistically significant association was also found between access to financial assistance and possession of prosthetic devices (*p ≤* 0.001). The majority (62.8%; *n* = 1899) of participants had no source of income and only 3.3% (*n* = 101) of the participants had a regular source of income. Possession of prosthesis and a source of income were significantly associated (*p ≤* 0.001).

**TABLE 2:** Socio-economic characteristics of participants with or without prosthetic devices.

Social characteristics	Possession of prosthesis				Total		*p*
	No		Yes				
	*n*	%	*n*	%	*N*	%	
**Employment**	-	-	-	-	-	-	< 0.001
Paid work	46	1.5	99	3.3	145	4.8	-
Self-employed	451	14.9	446	14.7	897	29.6	-
Unemployed	960	31.7	445	14.7	1405	46.4	-
Housekeeping	145	4.8	88	2.9	233	7.7	-
Student	103	3.4	83	2.7	186	6.1	-
Retired	79	2.6	12	0.4	91	3.0	-
Non-paid work and/or volunteering	8	0.3	12	0.4	20	0.7	-
Others	32	1.1	17	0.5	49	1.6	-
**Level of amputation**	-	-	-	-	-	-	< 0.001
Below knee	759	25.1	667	22.0	1426	47.1	-
Above knee	880	29.1	503	16.6	1383	45.7	-
Bilateral above amputation	14	0.5	1	0.0	15	0.5	-
Bilateral above and below knee amputation	8	0.3	8	0.3	16	0.5	-
Bilateral below knee amputation	9	0.3	2	0.1	11	0.4	-
Others	154	5.1	21	0.7	175	5.8	-
**Had dependents**	-	-	-	-	-	-	< 0.001
No	735	24.3	388	12.8	1123	37.1	-
Yes	1089	36.0	814	26.9	1903	62.9	-
**Had financial assistance**	-	-	-	-	-	-	< 0.001
No	1153	38.1	879	29.0	2032	67.2	-
Yes	671	22.2	323	10.7	994	32.8	-
**Source of income**	-	-	-	-	-	-	< 0.001
Regular	16	0.5	85	2.8	101	3.3	-
No source of income	1319	43.6	580	19.2	1899	62.9	-
Sometimes	489	16.2	537	17.7	1026	33.9	-

**Total**	**1824**	**60.3**	**1202**	**39.7**	**3026**	**100.0**	-

### Status of the prosthetic device

The functional status of participants’ prosthetic devices (Table 3) was highlighted as follows: 20.1% (*n* = 242) of them were in a good condition; among them, those with the transtibial 11.1% (*n* = 134) were in the majority. The study also found that 62.4% (*n* = 705) of the prosthetic devices needed repair and that 14.8% (*n* = 178) of prosthetic devices were completely damaged. There was no statistically significant association between the type of prosthesis and the status of the prosthesis (*p* = 0.604). A total of 3.5% (*n* = 162) of the prosthetic devices that were in good condition had been used for more than 12 months. Furthermore, 58.7% (*n* = 706) of those in need of repair had been also used for more than 12 months. A statistically significant association between the duration of using a prosthesis and the status of the prosthesis was found (*p ≤* 0.001). A total of 41.8% (*n* = 502) of participants with dependents had the most prosthetic devices that needed repair, although there was no statistically significant association between having dependents and the status of the prosthesis (*p* = 0.857).

**TABLE 3:** Status of prosthetic devices of participants.

Economic characteristics	Status of Prosthesis								Total		*p*
	In good condition		In use but needs repair		Broken and cannot be used		Never been used				
	*n*	%	*n*	%	*n*	%	*n*	%	*N*	%	
**Type of prosthesis**											0.604
Transtibial	134	11.1	427	35.5	97	8.1	14	1.2	672	55.9	
Transfemoral	106	8.8	311	25.9	80	6.7	17	1.4	514	42.8	
Both transtibial and transfemoral	1	0.1	3	0.2	1	0.1	0	0.0	5	0.4	
Others	1	0.1	9	0.7	0	0.0	1	0.1	11	0.9	
**Duration of using a prosthesis (in months)**											< 0.001
0–6	27	2.2	8	0.8	2	0.2	1	0.1	38	3.2	
7–12	53	4.4	36	3.0	7	0.6	5	0.4	101	8.4	
More than 12	162	13.5	706	58.7	169	14.1	26	2.2	1063	88.4	
**Having dependents**											0.857
No	78	6.5	248	20.6	64	5.3	10	0.8	400	33.3	
Yes	164	13.6	502	41.8	114	9.5	22	1.8	802	66.7	
**Source of income**											0.765
Regular	9	0.7	26	2.2	6	0.5	2	0.2	43	3.6	
No source of income	158	13.1	477	39.7	108	9.8	23	1.9	766	63.7	
Sometimes	75	6.2	247	20.5	64	5.3	7	0.6	393	32.7	
**Social class (Ubudehe)**											0.921
Category 1	144	12.0	425	35.4	95	7.9	19	1.6	683	56.8	
Category 2	82	6.8	267	22.2	67	5.6	11	0.9	427	35.5	
Category 3	16	1.3	58	4.8	16	1.3	2	0.2	92	7.7	

**Total**	**242**	**20.1**	**750**	**62.4**	**178**	**14.8**	**32**	**2.7**	**1202**	**100.0**	

The study further revealed that 39.7% (*n* = 477) of participants with no source of income had prosthetic devices that needed repair, while participants with an irregular source of income with prosthetic devices that needed repair were 20.5% (*n* = 247). A total of 35.4% (*n* = 425) and 22.2% (*n* = 267) of the prosthetic devices that needed repair were from Ubudehe Category 1 and 2, respectively. Category 1 had the most broken and/or damaged prosthetic devices; however, there was no statistically significant association between Socio-economic status and the status of the prosthesis (*p* = 0.921).

## Discussion

The purpose of this study was to establish the number of PLLA possessing or not prosthesis and/or mobility assistive devices and to determine their socio-economic profile in Rwanda. Studies have revealed that in low-income countries, the majority of PLLA have no mobility assistive devices such as prosthetic devices (De Witte et al. 2018). This was in line with findings from this study, which showed that 60.3% of PLLA did not have lower limb prosthetic devices. Almost similar results were also reported by the National Council of Persons with Disability in Rwanda, which indicated that 88% of persons with amputation needed prosthetic devices (Kidd & Kabare 2019).

The findings in this study further highlighted that among the persons with prosthetic devices 62.4% of the participants’ prosthetic devices were damaged, and therefore needed repair while 14.8% were completely damaged and needed replacement. These findings concur with the study carried out by Amosun, Mutimura and Frantz (2005) in Rwanda and by Magnusson and Ahlstrom (2017) in Malawi and Sierra Leone. This further demonstrates the increasing gap of service delivery for prosthetic devices, which may however also be worsened by the low socio-economic levels of PLLA, because they cannot afford the cost of repairing their devices. The low socio-economic levels of PLLA and the inability to afford the cost of repairing their prosthetic devices were highlighted in this study for Rwandans as well as in other low-income countries as indicated by Desmond et al. (2018). In Rwanda, repairs of the prosthesis are carried out at a cost although they are less expensive compared with manufacturing, and the process of repair goes through the same system because the devices are repaired at the same health facility where they are made and sometimes health facilities are not near their homes; therefore, this increases the cost and time.

This therefore emphasises that there is gap in acquiring prosthetic devices among person with LLA in Rwanda. A lack of mobility assistive devices or prosthesis has been highlighted elsewhere in low-income countries such as a study carried out in Nepal where prospects of independent lifestyle are minimum (Järnhammer et al. 2018; Wyss et al. 2015). The World Health Organization (WHO) is therefore advocating for countries to provide assistive mobility devices such as prosthesis to all those in need, and it goes further to request all countries to include assistive mobility devices on their list of essential products (Smith et al. 2018). While it is still a challenge, efforts have been made to put policies in place by both the government of Rwanda and other stakeholders such as charity and non-government organisations working with persons with disabilities in Rwanda to include prosthetic services on the insurance schemes, the government pays 90% of the cost of the assistive devices within government health facilities and the beneficially pays 10%. However, challenges in accessing prosthetic services are also linked to poverty among PLLA because the majority cannot afford the 10% cost of the prosthesis as well as the transport to these health facilities as highlighted in this study where the majority did not have any source of income or any financial assistance. Even though PLLA have other types of assistive devices such as crutches, it is thus obvious that without prosthetic devices, a person with LLA will have mobility challenges, hence leading to low socio-economic status highlighted by a number of studies (Lin & Wu 2014; Smith et al. 2018; Von Kaeppler et al. 2021).

The findings of this study found that males were in the majority with LLA (68.8%) compared with females. This study agrees with a systematic review by Godlwana et al. that there are high incidences of LLA among males than females (Godlwana, Nadasan & Puckree 2008). A systematic review carried out by Davie-smith et al. highlighted that high rates of amputations in high-income countries may be because of risk factors such as smoking and severe peripheral arterial disease (PAD) among males than in females (Davie-Smith et al. 2017) compared with traumatic injuries in low-income countries, for example, in a study carried out in Pakistan (Ahmad et al. 2019). More so, this may be attributed to the type of daily activities performed by men compared with women. Males in low income countries, Rwanda inclusive, are mostly engaged in hard labour such as farming, mining, cycling motorist as highlighted in the study carried out in Rwanda on road traffic accidents were male constituted 78% of the total accidents (Ahmad et al. 2019; Twagirayezu et al. 2008).

Rwanda is a low-income country with low literacy levels, and similar to many other low-income countries, the situation is the same for PLLA (Kidd & Kabare 2019). The majority of participants in this study either never attended school or attained primary-level education. However, the study conducted in Tanzania indicated different results from the current findings in Rwanda where the majority of participants had attained a high school level of education but still their level of education was lower compared with those in the developed countries (Von Kaeppler et al. 2021). The minimal formal education in low income countries affects PLLA in accessing employment the most because of limited employment skills, therefore they work as hard labourers. This study confirms that the majority of the participants were unemployed with no source of income to sustain their families. Consequently, without a source of income, PLLA were more likely to become poorer than other people in their communities, this is in agreement with the study carried out in Rwanda where persons with disability have less source of income (Kiregu et al. 2016; Sinha et al. 2011). However, this may also be because of a number of other factors other than the education level only. Factors, such as a lack of prosthesis, and many others can be explored more deeply in future studies in Rwanda.

Although the majority of participants reported that they were married and had families to take care of, most of them did not have prosthetic devices in addition to being unemployed. Therefore, this puts more burden on their well-being as well as that of their families, hence lowering their socio-economic status (Kiregu et al. 2016). The lack of prosthetic devices coupled with a lack of education is a limitation to the acquisition of the right skills for gainful employment.

Findings from this study further show that 81.5% of participants live in rural areas. This is different from the study performed in India where the majority of the amputees were living in towns and metropolitans (Sinha et al. 2011). The likely reason is that when individuals are amputated they find it costly to live in urban areas where the cost of living is higher compared with the rural areas, and therefore prefer to live in the rural areas where the cost of living is low (Kidd & Kabare 2019; Wekesa et al. 2013). Generally, the living conditions in rural areas are lower than urban areas in Rwanda however there are no basic services and infrastructure (Ayalon & Tesch-Römer 2018).

Furthermore, this study highlighted that 47.1% of participants had below the knee amputation. The results concur with two other studies, one carried out in Rwanda and the other in Malaysia, where there were more below the knee amputations than above the knee amputations and other types of lower amputations (Kidd & Kabare 2019; Razak et al. 2016). However, these findings were different from the study carried out in Nigeria where the majority were above the knee amputation (Agu & Ojiaku 2016). Studies have shown that when persons with below the knee amputations are given prosthetic devices, they get a quick recovery and re-integration into the community, therefore have high chances of improving their quality of life as compared to the ones with above the knee amputation (Knežević et al. 2015; Matos, Naves & De Araujo 2020). However, a study performed by Ng et al. in Brunei emphasised that below knee prosthesis guarantees physical functioning than the emotional well-being of PLLA (Ng et al. 2020).

Furthermore, from the results, 62.8% of participants had no source of income yet had families and dependents to take care of. The findings concur with the study carried out in Malaysia where the majority of participants were in low-income classes (Razak et al. 2016). However, the number of participants who had prostheses and a source of income were slightly higher than the participants with no source of income. This may be the reason why there was a statistically significant association between possession of a prosthesis and a source of income. The results complement other studies that have highlighted that possession of prostheses contributed to a better quality of life for PLLA than those without prosthesis (Razak et al. 2016).

The majority of participants’ lack of a source of income in this study was the reason for most of them to be in Categories 1 and 2 of ‘Ubudehe’ social classification. This shows that PLLA are among the poorest. Although the majority did not have any source of income, a small number of 32.8% of participants were given financial assistance by the government for their daily upkeep but still the assistance was not enough to meet their needs. The universal health insurances cover 90% of the cost of the devices. Yet, most of the PLLA are not able to afford the remaining 10% of the cost of the prosthetic device because they have no source of income and no government assistance, hence making their living conditions worse and their socio-economic status poorer.

This research has highlighted that for PLLA to improve their quality of life, they must have mobility assistive devices such as prosthesis (Magnusson & Ahlstrom 2017). Generally, prosthetic devices are more expensive in Rwanda, but the government of Rwanda has included them on the list of medical equipment that is covered by all health insurances in Rwanda. However, not everybody can afford to pay for the individual contribution. Therefore, it is argued that stakeholders such as faith-based organisations and charity non-government organisations (NGOs) should work hand in hand with the government to provide affordable prosthetic devices to PLLA (Maclachlan et al. 2018).

### Implications

The study’s findings have highlighted the gap in accessibility and affordability of prosthetic devices to PLLA in Rwanda as the majority of participants in the study did not have them. Regarding the socio-economic status of PLLA, it was highlighted that the majority of them were among the poorest, in Category 1 as classified by the ‘Ubudehe’ classification in Rwanda. The findings may provide evidence to the government and stakeholders that may contribute to better planning and decision making towards the improvement of the welfare of PLLA. The study’s findings may inform policymakers and other stakeholders to formulate policies that may improve the accessibility and affordability of prosthetic devices for PLLA. This will be a basis for the improvement of their mobility and addressing their socio-economic challenges, hence influencing their socio-economic status. The study’s findings may also be a basis for further research on the quality of life for PLLA and re-integration into the community.

### Limitation

The limitations of this study were the following: the coronavirus disease 2019 pandemic led to travel restrictions in the country and limited funding during data collection.

The coronavirus disease 2019 COVID-19 Standard Operating Procedures (SOPs) limited personal contacts with the participants as well as paperwork like the use of questionnaires.

## Conclusion

Based on the findings from this study, it is evident that the majority of PLLA in Rwanda do not have prosthetic devices, or even those who have them, are damaged and in need of repair to remain functional. As the majority of PLLA do not have a source of income and are poor, hence worsening their socio-economic status, it is therefore difficult to afford prosthetic devices. Therefore, the government’s collaboration with other stakeholders such as charity and faith-based NGOs working with persons with disabilities should put up mechanisms and/or strategies to make the devices accessible and affordable to PLLA.

## References

[CIT0001] Agu, T.C. & Ojiaku, M.E., 2016, ‘The indications for major limb amputations: 8 years retrospective study in a private orthopaedic and trauma centre in the south-east Nigeria’, *Journal of Clinical Orthopaedics and Trauma* 7(4), 242–247. 10.1016/j.jcot.2016.03.006 27857497 PMC5106472

[CIT0002] Ahmad, A., Ashfaq, O., Akhtar, N., Rana, T. & Gul, M., 2019, ‘Causes of lower limb amputation in patients registered at Pakistan Institute of Prosthetic and Orthotic Sciences Peshawar-Pakistan’, *Khyber Medical University Journal* 11(1), 41–44. 10.35845/kmuj.2019.18096

[CIT0003] Amosun, S.L., Mutimura, E. & Frantz, J.M., 2005, ‘Health promotion needs of physically disabled individuals with lower limb amputation in Rwanda’, *Disability and Rehabilitation* 27(14), 837–847. 10.1080/09638280400018676 16096236

[CIT0004] Anderson, S., Kaiser Gladwin, K. & Mayo, N., 2016, ‘Considering material culture in assessing assistive devices: “Breaking up the rhythm”’, *Societies* 6(2), 14. 10.3390/soc6020014

[CIT0005] Asano, M., Rushton, P., Miller, W.C. & Deathe, B.A., 2008, ‘Predictors of quality of life among individuals who have a lower limb amputation’, *Prosthetics and Orthotics International* 32(2), 231–243. 10.1080/03093640802024955 18569891

[CIT0006] Ayalon, L. & Tesch-Römer, C., 2018, ‘Understanding the experience of elderly in rural areas Rwanda’, in P. Maharaj (ed.), *International perspectives on aging, innovation in aging*, Springer, Durban.

[CIT0007] Davie-Smith, F., Paul, L., Nicholls, N., Stuart, W.P. & Kennon, B., 2017, ‘The impact of gender, level of amputation and diabetes on prosthetic fit rates following major lower extremity amputation’, *Prosthetics and Orthotics International* 41(1), 19–25. 10.1177/0309364616628341 26850990 PMC5302066

[CIT0008] Desmond, D., Layton, N., Bentley, J., Boot, F.H., Borg, J., Dhungana, B.M. et al., 2018, ‘Disability and rehabilitation: Assistive technology and people: A position paper from the first global research, innovation and education on assistive technology (GREAT) summit’, *Disability and Rehabilitation: Assistive Technology* 13(5), 437–444. 10.1080/17483107.2018.1471169 29772940

[CIT0009] De Witte, L., Steel, E., Gupta, S., Ramos, V.D. & Roentgen, U., 2018, ‘Assistive technology provision: Towards an international framework for assuring availability and accessibility of affordable high-quality assistive technology’, *Disability and Rehabilitation: Assistive Technology* 13(5), 467–472. 10.1080/17483107.2018.1470264 29741965

[CIT0010] Gallagher, P. & MacLachlan, M., 2000, ‘Development and psychometric evaluation of the trinity amputation and prosthesis experience scales (TAPES)’, *Rehabilitation Psychology* 45(2), 130–154. 10.1037/0090-5550.45.2.130

[CIT0011] Godlwana, L., Nadasan, T. & Puckree, T., 2008, ‘Global trends in incidence of lower limb amputation’, *SA Journal of Physiotherapy* 64(8), 1–5. 10.4102/sajp.v64i1.93

[CIT0012] Järnhammer, A., Andersson, B., Wagle, P.R. & Magnusson, L., 2018, ‘Living as a person using a lower-limb prosthesis in Nepal’, *Disability and Rehabilitation* 40(12), 1426–1433. 10.1080/09638288.2017.1300331 28320228

[CIT0013] Kidd, S. & Kabare, K., 2019, ‘Social protection and disability in Rwanda’, August, pp. 1–56, viewed 05 March 2022, from www.developmentpathways.co.uk.

[CIT0014] Kiregu, J., Murindahabi, N.K., Tumusiime, D., Thomson, D.R., Hedt-Gauthier, B.L. & Ahayo, A., 2016, ‘Socioeconomics and major disabilities: Characteristics of working-age adults in Rwanda’, *PLoS One* 11(4), e0153741. 10.1371/journal.pone.0153741 27101377 PMC4839600

[CIT0015] Knežević, A., Salamon, T., Milankov, M., Ninković, S., Jeremić Knežević, M. & Tomašević Todorović, S., 2015, ‘Assessment of quality of life in patients after lower limb amputation’, *Medicinski Pregled* 68(3–4), 103–108. 10.2298/MPNS1504103K 26214989

[CIT0016] Lin, I. & Wu, H., 2014, ‘Activity limitations, use of assistive devices or personal help, and well-being: Variation by education’, *The Journals of Gerontology: Series B, Psychological Sciences and Social Sciences* 69(Suppl 1), 16–25. 10.1093/geronb/gbu115 PMC4303070 25342819

[CIT0017] Maclachlan, M., Banes, D., Bell, D., Borg, J., Donnelly, B., Fembek, M. et al., 2018, ‘Disability and rehabilitation: Assistive technology policy: A position paper from the first global research, innovation, and education on assistive technology (GREAT) summit’, *Disability and Rehabilitation: Assistive Technology* 13(5), 454–466. 10.1080/17483107.2018.1468496 29790393

[CIT0018] Magnusson, L. & Ahlstrom, G., 2017, ‘Patients’ satisfaction with lower-limb prosthetic and orthotic devices and service delivery in Sierra Leone and Malawi’, *BMC Health Services Research* 17, 102. 10.1186/s12913-017-2044-3 28143549 PMC5286686

[CIT0019] Matos, D.R., Naves, J.F. & De Araujo, T.C.C.F., 2020, ‘Quality of life of patients with lower limb amputation with prostheses’, *Estudos de Psicologia (Campinas)* 37, 1–12. 10.1590/1982-0275202037e190047

[CIT0020] Matter, R.A. & Eide, A.H., 2018, ‘Access to assistive technology in two Southern African countries’, *BMC Health Services Research* 18, 792. 10.1186/s12913-018-3605-9 30340484 PMC6194741

[CIT0021] National Institute of Statistics of Rwanda, 2014, ‘Socio-economic characteristics of persons with disabilities: Thematic report’, p. 114, Government of Rwanda, Ministry of finance and economic planning, Kigali Rwanda.

[CIT0022] Ng, S.S., Naing, L., Idris, F.I. & Pande, K., 2020, ‘What is the quality of life of transtibial amputees in Brunei darussalam?’, *Malaysian Orthopaedic Journal* 14(2), 39–46. 10.5704/MOJ.2007.009 32983376 PMC7513655

[CIT0023] Razak, M.M.A., Tauhid, M.Z., Yasin, N.F. & Hanapiah, F.A., 2016, ‘Quality of life among lower limb amputees in Malaysia’, *Procedia – Social and Behavioral Sciences* 222, *African Journal of Disability* 2(1), 450–457. 10.1016/j.sbspro.2016.05.135

[CIT0024] Rhoda, A.J. & Eide, A.H., 2009, ‘Barriers to and facilitators of rehabilitation services for people with physical disabilities: A systematic review’, pp. 1–6. 10.4102/ajod.v2i1.22 PMC5442576 28729982

[CIT0025] Sangam, S., Naveed, A., Athar, M., Prathyusha, P., Moulika, S. & Lakshmi, S., 2015, ‘A study on functional measures in patients with stroke’, *International Journal of Health Sciences and Research* 5(1), 156–164.

[CIT0026] Scorza, P., Stevenson, A., Canino, G., Mushashi, C., Kanyanganzi, F. & Munyanah, M., 2013, ‘Validation of the “World Health Organization disability assessment schedule for children, WHODAS-Child” in Rwanda’, *PLoS One* 8(3), e57725. 10.1371/journal.pone.0057725 23505437 PMC3591425

[CIT0027] Sinha, R., Van Den Heuvel, W.J.A. & Arokiasamy, P., 2011, ‘Factors affecting quality of life in lower limb amputees’, *Prosthetics and Orthotics International* 35(1), 90–96. 10.1177/0309364610397087 21515894

[CIT0028] Smith, E.M., Gowran, R.J., Mannan, H., Donnelly, B., Alvarez, L., Bell, D. et al., 2018a, ‘Disability and rehabilitation: Assistive technology enabling appropriate personnel skill-mix for progressive realization of equitable access to assistive technology’, *Disability and Rehabilitation: Assistive Technology* 13(5), 445–453. 10.1080/17483107.2018.1470683 29772939

[CIT0029] Smith, R.O., Scherer, M.J., Cooper, R., Bell, D., Hobbs, D.A., Pettersson, C. et al., 2018b, ‘Assistive technology products: A position paper from the first global research, innovation, and education on assistive technology (GREAT) summit’, *Disability and Rehabilitation: Assistive Technology*, 13(5), 473–485. 10.1080/17483107.2018.1473895 29873268

[CIT0030] Twagirayezu, E., Teteli, R., Bonane, A. & Rugwizangoga, E., 2008, ‘Road traffic injuries at Kigali University Central Teaching Hospital, Rwanda’, *East and Central African Journal of Surgery* 13(1), 73–76.

[CIT0031] Üstün, T.B., Chatterji, S., Kostanjsek, N., Rehm, J., Kennedy, C., Epping-Jordan, J. et al., 2010, ‘Developing the World Health Organization disability assessment schedule 2.0’, *Bulletin of the World Health Organization* 88(11), 815–823. 10.2471/BLT.09.067231 21076562 PMC2971503

[CIT0032] Van Twillert, S., Stuive, I., Geertzen, J.H., Postema, K. & Lettinga, A.T., 2014, ‘Functional performance, participation and autonomy after discharge from prosthetic rehabilitation: Barriers, facilitators and outcomes’, *Journal of Rehabilitation Medicine* 46(9), 915–923. 10.2340/16501977-1846 25074343

[CIT0033] Von Kaeppler, E.P., Hetherington, A., Donnelley, C., Ali, S., Shirley, C., Challa, S. et al., 2021, ‘Impact of prostheses on quality of life and functional status of transfemoral amputees in Tanzania’, *African Journal of Disability* 10, 1–10. 10.4102/ajod.v10i0.839 PMC8517763 34692432

[CIT0034] Wekesa, V.D., Ogengo, J.A., Elbusaidy, H., Siongei, C.V. & Iwaret, M., 2013, ‘Demograpics of patients admitted with traumatic intracranial bleeds in Kenyatta National Hospital in Nairobi, Kenya’, *East & Central African Journal of Surgery* 18(3), 67–70.

[CIT0035] Wyss, D., Lindsay, S., Cleghorn, W. & Andrysek, J., 2015, ‘Priorities in lower limb prosthetic service delivery based on an international survey of prosthetists in low- and high-income countries’, *Prosthetics and Orthotics International* 39(2), 102–111. 10.1177/0309364613513824 24335154

